# Hydrogen sulfide as a therapeutic option for the treatment of Duchenne muscular dystrophy and other muscle-related diseases

**DOI:** 10.1007/s00018-022-04636-0

**Published:** 2022-11-28

**Authors:** Katarzyna Kaziród, Małgorzata Myszka, Józef Dulak, Agnieszka Łoboda

**Affiliations:** Department of Medical Biotechnology, Faculty of Biochemistry, Biophysics and Biotechnology, Jagiellonian University in Krakow, Gronostajowa 7, 30-387 Kraków, Poland

**Keywords:** Duchenne muscular dystrophy, Hydrogen sulfide, Inflammation, H_2_S donors, Skeletal muscles

## Abstract

Hydrogen sulfide (H_2_S) has been known for years as a poisoning gas and until recently evoked mostly negative associations. However, the discovery of its gasotransmitter functions suggested its contribution to various physiological and pathological processes. Although H_2_S has been found to exert cytoprotective effects through modulation of antioxidant, anti-inflammatory, anti-apoptotic, and pro-angiogenic responses in a variety of conditions, its role in the pathophysiology of skeletal muscles has not been broadly elucidated so far. The classical example of muscle-related disorders is Duchenne muscular dystrophy (DMD), the most common and severe type of muscular dystrophy. Mutations in the *DMD* gene that encodes dystrophin, a cytoskeletal protein that protects muscle fibers from contraction-induced damage, lead to prominent dysfunctions in the structure and functions of the skeletal muscle. However, the main cause of death is associated with cardiorespiratory failure, and DMD remains an incurable disease. Taking into account a wide range of physiological functions of H_2_S and recent literature data on its possible protective role in DMD, we focused on the description of the ‘old’ and ‘new’ functions of H_2_S, especially in muscle pathophysiology. Although the number of studies showing its essential regulatory action in dystrophic muscles is still limited, we propose that H_2_S-based therapy has the potential to attenuate the progression of DMD and other muscle-related disorders.

## Duchenne muscular dystrophy (DMD): an overview

Duchenne muscular dystrophy (DMD) represents one of the most common, severe, and lethal types of dystrophinopathies, which are caused by mutations in the X-linked *DMD* gene that encodes dystrophin, a structural muscle protein. The clinical presentation of DMD was first described in the 1850s–1860s; however, the first fragments of the *DMD* gene cDNA were identified more than one hundred years later (for references, see: [1]). DMD is characterized by progressive weakness of the skeletal and cardiac muscle due to muscular damage and degeneration. Patients suffer from motor delays, loss of ambulation, cardiomyopathy, and respiratory impairment [2]. The first symptoms of the disease appear around 2–3 years of age, including frequent falls, difficulty getting up from the floor, the need for help with the hands to stand up (Gower’s sign), or problems with running and climbing stairs. Rapid progression of the disease is observed around the age of 7 with later motor disability, which requires wheelchair use around the age of 12. This is followed by progressive heart problems that require pharmacological treatment, mechanical cardiac support and surgical solutions, and respiratory failure that leads to assisted ventilation.

**Fig. 1:**
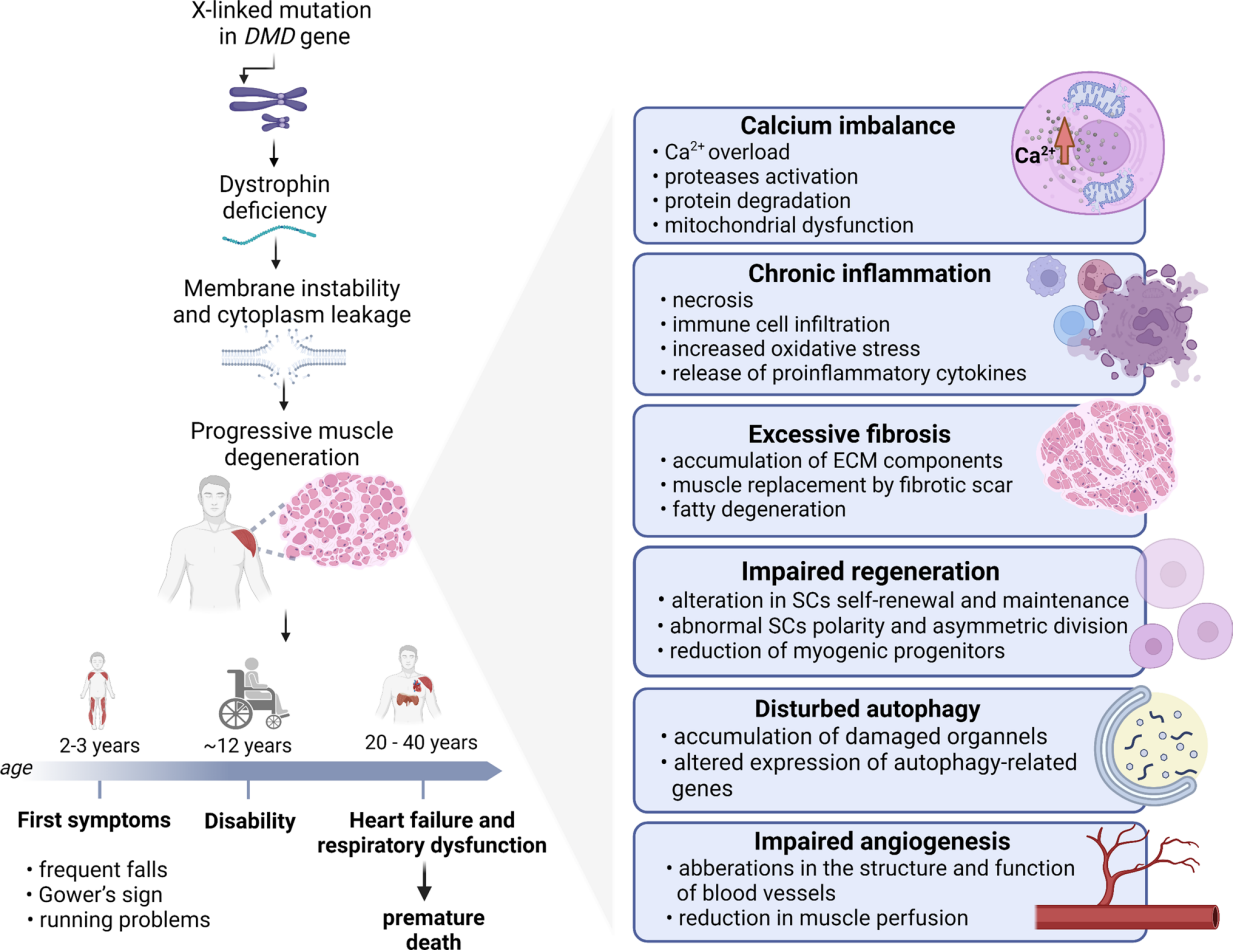
Progression of DMD and its complications. Duchenne muscular dystrophy (DMD) is caused by an X-linked mutation in the *DMD* gene. Dystrophin deficiency leads to membrane instability and cytoplasm leakage, leading to progressive muscle degeneration. The main hallmarks of DMD are calcium imbalance, chronic inflammation, excessive fibrosis, impaired regeneration, altered autophagy, and dysregulated angiogenesis. The first symptoms of DMD occur around age 2–3 and manifest themselves as frequent falls, Gower's sign, and running problems. Progressive muscle weakness results in disability around the age of 12. This is followed by heart failure and respiratory dysfunction that leads to premature death in the second to fourth decades of life

Respiratory dysfunction and cardiological complications are among the most devastating effects of the disease in patients with DMD, contributing to their morbidity and mortality. Progressive sequelae of DMD, such as scoliosis and kyphosis, result in altered lung function, and respiratory muscle weakness can lead to decreased ventilation capacity and pulmonary infections. Improved survival of patients with respiratory failure was achieved thanks to the introduction of noninvasive nocturnal mechanical ventilation; however, enhanced survival and extension of life expectancy of dystrophic patients result in increased DMD-related cardiomyopathy [3, 4]. Notably, heart problems represent an alternative cause of death associated with DMD and do not correlate with the extent of skeletal muscle degeneration [5]. The first incidence of cardiomyopathy occurs at the age of 6 years. Symptoms manifest themselves in dilated cardiomyopathy, which progresses to end-stage heart failure with accompanying supraventricular and ventricular arrhythmias [6]. Unfortunately, until now, DMD is an incurable disease, and even with optimal care, patients die between the second and fourth decade of life [7, 8] (Fig. 1).

## *DMD* gene, dystrophin, and dystrophin–glycoprotein complex (DGC)

The gene encoding dystrophin represents one of the largest human genes, spanning more than 2200 kb (0.1% of the whole genome) [9]. It contains 79 exons, and the most common mutation causing DMD is the deletion of one or more exons, which constitutes about 60–70% of all DMD cases, while point mutations account for 26% of cases and exonic duplication cause another 10–15%. Missense mutations, splice mutations, and subexonic insertions or deletions are also responsible for some cases. Importantly, mutations that cause DMD disrupt the protein reading frame, causing premature stop codons, resulting in no or insufficient protein production [10].

DMD affects around 1:5000 male newborns; however, it also rarely occurs in women (less than one per million) with Turner syndrome, biallelic *DMD* mutations, or translocations involving *DMD*. In most cases, female carriers are usually asymptomatic, however, some manifest symptoms resembling the milder type of dystrophinopathy, Becker muscular dystrophy (BMD) [8]. BMD is also caused by mutations in the gene encoding dystrophin; however, these mutations (in-frame deletions) maintain the correct reading frame. In contrast to DMD, this leads to the production of a shorter, partially functional form of *DMD* products, which are internally truncated and expressed at lower levels compared to healthy individuals [10].

Due to the complex structure of the *DMD* gene (for example, two polyadenylation sites and seven independent tissue-specific promoters), alternative splicing gives the number of various DMD isoforms, different in size and tissue distribution [11]. The full-length muscular isoform of dystrophin with a molecular weight of 427 kDa (Dp427m) is composed of four main functional domains, an N-terminal actin-binding domain (ABD), a central coiled-coil segment of 24 spectrin-like repeats, and 4 hinge domains that form so so-called rod domain (ROD), cysteine-rich domain (CRD), and C-terminal domain (CTD) [12]. Almost 50% of the mutations that occur in patients with DMD are located within the ABD fragment, to a lesser extent in the CRD and ROD, and just over 10% of the mutations are associated with the CTD [13].

Dystrophin is located on the cytoplasmic side of the muscle and the cardiac sarcolemma. The important function of the protein is to maintain the integrity of the myofiber plasma membrane during force generation, providing mechanical stabilization. Dystrophin is one of the key proteins that forms the dystrophin-associated protein complex (DAPC), also known as the dystrophin–glycoprotein complex (DGC), and provides linkage via the N-terminus and C-terminus (binding to the transmembrane dystroglycan complex) between the actin cytoskeleton and the extracellular matrix, respectively [10, 14]. Furthermore, DGC plays a signaling role by controlling mechanical force transduction and cell adhesion and functions as a scaffold for signaling proteins [15]. DGC regulates muscle cell NO signaling and maintains optimal activity of neuronal nitric oxide synthase (nNOS) and calcium (Ca^2+^) homeostasis [4].

## Consequences of dystrophin deficiency and pathological hallmarks of DMD

Although dystrophin represents only approximately 0.002% of total muscle protein, its lack causes enormous changes in skeletal and cardiac muscle functions such as inflammation, fibrosis, and degeneration [16] (Fig. 1). Increased permeability of the cell membrane allows larger proteins, including muscle creatine kinase (CK) and lactate dehydrogenase (LDH), to enter the circulation. In fact, serum CK and LDH can be used as biomarkers of DMD [17]. Furthermore, under dystrophin-deficient conditions, an increased Ca^2+^ influx and Ca^2+^ overload were demonstrated in both human and animal models of the disease [18, 19]. Consequently, protein degradation and activation of calcium-dependent proteases such as calpains and numerous chemokines and cytokines occur [20]. This induces inflammation by activating the activation of the nuclear factor kappa-light-chain-enhancer of activated B cells (NF-κB), which triggers transcription of inducible nitric oxide synthase (iNOS) [21]. Calcium imbalance is also closely related to mitochondrial function. Increased intracellular Ca^2+^ leads to upregulation in mitochondrial Ca^2+^ uptake, which further results in accelerated mitochondrial production of reactive oxygen species (ROS). This causes depolarization of the mitochondrial membrane and the opening of the mitochondrial permeability transition pore (MPTP) and decreased ATP production. It should be noted that an imbalance in calcium homeostasis has a more detrimental effect on cardiac cells compared to skeletal muscle [21]; however, in both cell types these disturbances result in cell death through necrosis and apoptosis [20].

Necrosis is the first step in stimulating an inflammatory response, and chronic inflammation and infiltration of immune cells are one of the main hallmarks of DMD. Several mechanisms contribute to these processes, but the innate immune system is first activated by membrane instability and cytoplasm leakage. Destroyed fibers release damage-associated molecular patterns (DAMPs), such as nucleic acids, ROS, and heat shock proteins (HSPs), and activate toll-like receptors (TLRs). Activation of TLRs and the interleukin-1 receptor (IL-1R) initiates a pro-inflammatory signaling cascade, including activation of the myeloid differentiation primary response 88/IL-1R-associated kinase (MyD88/IRAK) pathway. This, in turn, leads to stimulation of mitogen-activated protein kinases (MAPKs) and NF-κB [22]. Dystrophin-deficient muscles are infiltrated by immune cells such as macrophages, neutrophils, and mast cells. Furthermore, in DMD muscles, an elevated level of eosinophils is also present in the early stage of the disease. Among all leukocytes, myeloid cells are the most abundant immune cells that infiltrate dystrophin-deficient muscles; however, lymphoid cells, such as cytotoxic T-lymphocytes, are also present and contribute to the cytolytic and immunomodulatory effect [23]. Self-sustaining activation of the innate immune response leads to the constant release of pro-inflammatory cytokines including IL-1β, tumor necrosis factor-α (TNF-α), interferon-γ (IFN-γ), IL-6, induction of constitutive expression of MHC class I and II in muscle cells, and recruitment of T and B cells, which generates an adaptive immune response [24].

The diverse nature of macrophages contributes to the progression of DMD. In the early stage of the disease, Ly6C^pos^ pro-inflammatory macrophages promote muscle lesions due to their NO-mediated cytolytic capacity. Later, during the progression of the disease, IL-10-producing cells, such as regulatory T cells (T_regs_), induce alternative activation of Ly6C^neg^ anti-inflammatory macrophages. It leads to the deactivation of the inflammatory response and tissue repair [24]. This shows the complexity of the immune response in the course of DMD with repeated cycles of inflammation, damage, and activation of compensation mechanisms to counteract disease progression. In addition, macrophages also release pro-fibrotic molecules such as transforming growth factor-β (TGF-β), that is crucial in the initiation of the fibrotic process [24, 25]. Fibroblasts activated by TGF-β produce extracellular matrix (ECM) components and ECM-remodeling factors (collagens, fibronectin, metalloproteinases) [26]. As a result of chronic injury, persistent inflammation, and the presence of macrophages, an excessive amount of ECM components accumulate in muscle tissue. This inhibits myogenic repair and contributes to muscle replacement by the fibrotic scar [27]. Notably, a different, more rigid structure of the fibrotic tissue compared to the muscles affects the efficiency of muscle contraction. During this time, fibrotic tissue can also be replaced by adipocytes (fatty degeneration) [27, 28].

Myonecrosis, increased inflammation, oxidative stress, and progressive fibrosis alter the myogenesis and regeneration process in dystrophic muscles, which leads to a subsequent loss of muscle tissues [29]. Impaired regeneration in DMD muscles is associated with an inefficient generation of myogenic progenitors. Notably, dystrophin is expressed in muscle stem cells, satellite cells (SCs), defined as CD45^−^CD31^−^α7-integrin^+^Sca-1^−^ cells expressing Pax7, the canonical marker that drives their survival and proper functioning [30]. In SCs, dystrophin regulates their polarity and asymmetric division [31], and, interestingly, both an increase in their number [32], and their exhaustion due to telomere shortening [33] under dystrophic conditions have been shown. Despite an increased quantity of SCs in DMD muscles, the absence of dystrophin alters their self-renewal and maintenance, due to the decreased expression of asymmetric divisions-regulating microtubule affinity-regulating kinase 2 (MARK2) [31]. Our results, obtained in the *mdx* mouse model, also indicate a higher number of Pax7^+^ SCs in dystrophin-deficient mice, suggesting a lack of association between defects in the regenerative potential of SCs and their number [34–36]. It should be noted that the functioning of SCs is also regulated by infiltrating macrophages. Pro-inflammatory macrophages promote SCs proliferation, while anti-inflammatory macrophages favor their differentiation and fusion. Under dystrophic conditions, the increased and persistent presence of different macrophage phenotypes may contribute to alterations in SC function [26].

Recent studies have also shown that impaired autophagy is another hallmark of DMD and can be considered a new therapeutic target [37]. Lack of dystrophin leads to increased activation of Akt and mTOR, negative autophagy regulators [37, 38]. Furthermore, in dystrophin-deficient SCs enhanced phosphorylation and decreased nuclear translocation of coactivator-associated arginine methyltransferase 1 (CARM1) are observed. This not only leads to reduced transcription of Myf5 and other Pax7 target genes, resulting in impaired function of satellite cells, but may also affect the autophagy process [39]. CARM1 regulates autophagy in the adenosine 5′-monophosphate-activated protein kinase (AMPK)-dependent way [40]; therefore, its increased phosphorylation in DMD may be responsible for the inability to properly activate autophagy. Insufficient autophagy causes the accumulation of damaged organelles and protein aggregates that affects proper muscle repair [41]. Under dystrophic conditions, the gene expression of many proteins that participate in the autophagy process is dysregulated. We have found a decrease in the mRNA level of beclin-1 (*Becn1*), autophagy-related genes 5 and 7 (*Atg5*, *Atg7)*, and lysosomal-associated membrane protein 1 (*Lamp1*) in muscles from *mdx* mice [42]. The conversion of the soluble form of the microtubule-associated protein 1 light chain 3 (LC3-I) to lipid-bound LC3-II contributes to the formation of an autophagosome and is necessary, but not sufficient, to trigger cell autophagy [43]. A decrease in the level of the autophagy marker LC3-II was also observed in the DMD muscles [37]. There are also some studies suggesting activation of autophagy under dystrophic conditions, but this can be affected by age and progression of the disease [44, 45].

Furthermore, defective mitochondria-specific autophagy, mitophagy was found in dystrophic muscles. The mechanism may involve dysregulation of the PINK1 (PTEN-induced kinase-1)/PARKIN (Parkinson juvenile disease protein-2) pathway [42, 46]. The decrease in critical mitophagy-related factors, such as PINK1, PARK2, and BNIP3, has been demonstrated in dystrophic patients and various animal models of DMD (mice and worms) [42, 47]. On the other hand, the use of the mitophagy activator urolithin A alleviated the symptoms of DMD [47]. Kuno et al. demonstrated the beneficial cardioprotective effects of resveratrol in *mdx* mice, which was the consequence of the reactivation of defective mitophagy [48]. Similarly, activation of AMPK, the regulator of the autophagy-mitophagy pathway, improved the contractile functions of the dystrophic diaphragm by improving mitochondrial integrity [49]. Taken together, many studies have shown dysregulated autophagy and mitophagy in DMD; however, due to the complex nature of this process, more studies are warranted.

Angiogenesis, a process of the formation of new blood vessels, has also been suggested to be affected under dystrophic conditions (reviewed in: [50]). Importantly, dystrophin is expressed in vascular smooth muscle cells [51, 52] and, as mentioned above, in SCs that are close to the capillaries and secrete the key angiogenic factor, vascular endothelial growth factor (VEGF). Furthermore, the presence of dystrophin was suggested in endothelial cells [53, 54]; however, these results need further confirmation, as endothelial cell fractions instead of pure endothelial cells have been used in the above studies. Nevertheless, the absence of dystrophin can cause aberrations in the structure and functions of blood vessels and impaired angiogenesis in an age-dependent manner [50, 55, 56]. In DMD-affected muscles, a decrease in VEGF expression was observed in our studies [34–36, 56] and an increase in its level was proposed as a strategy that could exert a beneficial effect on the pathology of DMD (reviewed in: [50]).

### Treatment of Duchenne muscular dystrophy—possibilities and limitations

**Fig. 2:**
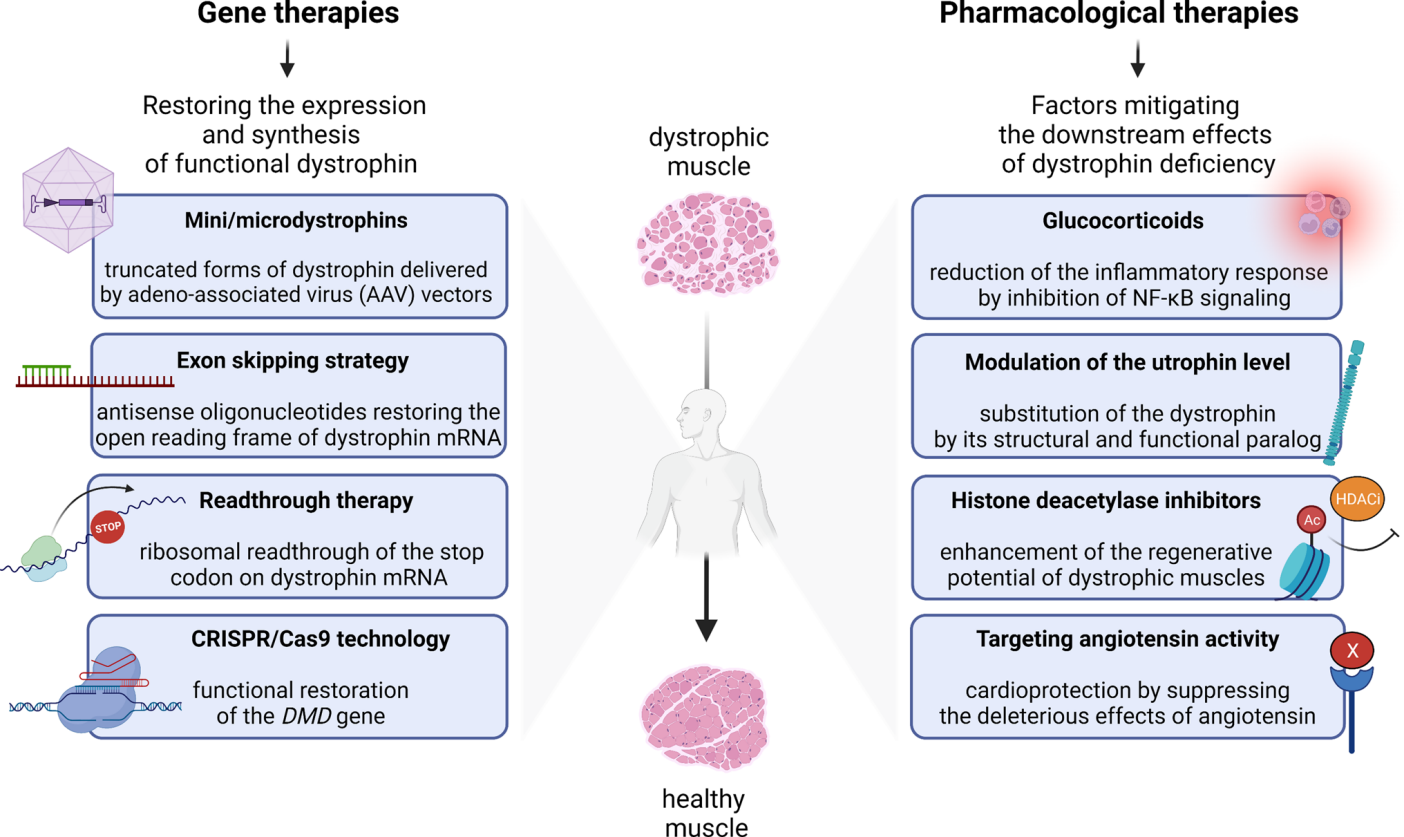
Examples of different therapeutic approaches for DMD treatment. Among the main therapeutic approaches for DMD are genetic and pharmacological therapies. Genetic therapies focus on restoring the expression and synthesis of functional dystrophin through the mini/micro-dystrophin approach, the exon skipping strategy, readthrough therapy, and the CRISPR/Cas9 technology. On the other hand, pharmacological therapies use factors such as glucocorticoids, modulators of utrophin level, histone deacetylase inhibitors, and compounds targeting angiotensin activity that mitigate the downstream effects of dystrophin deficiency

Despite extensive research on the molecular mechanisms of DMD, it remains an incurable and fatal disease. However, pharmacological, gene, and cell therapies, aimed at counteracting the processes that contribute to disease progression (described above) or the restoration of functional dystrophin, are currently being investigated (reviewed in [4]) (Fig. 2). Although some approaches are extremely promising, their widespread clinical application may be a matter of the distant future, also because of the drug-specific limitations. Combinatory therapies that simultaneously use strategies to target the cause of the disease and mitigate the secondary effects of the absence of dystrophin can have additive therapeutic benefits [57].

#### Gene therapies

There are several possibilities to restore the expression and synthesis of functional dystrophin through gene delivery; unfortunately, most viral vectors, apart from adeno-associated virus (AAV) vectors, do not infect myocytes with satisfactory efficiency. Although mature muscle cells can be targeted by AAV, discrepant data has been published on the ability of these vectors to transduce myogenic stem cells. Arnett et al. demonstrated that quiescent SCs are resistant to transduction in vivo in adult mice [58]. In contrast, Tabebordbar et al. were able to show that dystrophic SCs are transduced after systemic delivery of AAV9-*Dmd* CRISPR (AAV serotype 9) that leads to the restoration of the dystrophin reading frame [59]. Furthermore, further studies confirmed the ability of transduction and gene editing in SCs after AAV9 delivery of CRISPR-Cas9 components, albeit with rather a low efficiency [60].

More studies are needed to analyze in-depth, for example, the role of the satellite cell niche on SCs transduction and to increase efficiency by testing other muscle-specific promoters instead of the commonly used muscle creatine kinase 8 (CK8e) promoter, which is not highly active in resident SCs [60]. It should be noted that the AAV capacity is not sufficient to accommodate a complete dystrophin construct, and these vectors also cause an inflammatory response. However, much effort has been made to improve genetic methods for DMD treatment. Current approaches can be classified into four main groups, including (1) mini/micro-dystrophin (truncated forms of dystrophin that contain only the domains necessary for their function) through AAVs; (2) exon skipping strategy using antisense oligonucleotides (AONs) sequences to restore the open reading frame of dystrophin mRNA; (3) readthrough therapy related to the presence of nonsense mutations, and (4) the CRISPR/Cas9 gene-editing system to repair mutations in the *DMD* gene (summarized in: [4]).

It should be emphasized that all strategies aimed at restoring the expression and synthesis of functional dystrophin are challenging both from a technical point of view (e.g., the optimal route of administration must be selected to ensure the production of dystrophin not only in skeletal muscles but also in the heart) and from a biological point of view (e.g., to limit the risk of an immune response to dystrophin and/or AAV vectors). Another important issue is the estimation of the minimal level of dystrophin required to achieve a therapeutic effect [61].

Gene transfer of the full-length dystrophin coding sequence is technically difficult, as AAV vectors have a small packaging capacity (around 5 kb). Due to this limitation, truncated forms of dystrophin, the so-called mini/micro-dystrophin, have been generated, containing only the domains necessary for their function (ABD, CRD, and CTD, with the versatile length of the ROD structure) [62–64]. Despite positive effects in animal studies, the clinical efficacy of this strategy was not sufficient and problems with a very low level of micro-dystrophin in the muscles and an unsatisfactory transduction efficiency of the diaphragm and particularly the heart were evident [65]. Positive effects, observed in some clinical studies [66], can also be related to concomitant treatment with a high dose of corticosteroids, necessary to halt the immune response against the viral vector.

To overcome immune reactions generated by the newly produced dystrophin protein, therapy with utrophin, structural, and functional dystrophin paralog has been proposed. Like dystrophin, utrophin interacts with the dystrophin-associated protein complex to link the actin cytoskeleton to the extracellular matrix [67]. However, its expression in the extrajunctional sarcolemma declines after birth and, despite high expression during fetal development, it is replaced by dystrophin in adults [68, 69]. Therefore, stimulation of utrophin expression appeared to be an attractive strategy for treating dystrophic patients. However, although utrophin and dystrophin share many similarities, they also differ in some properties and functions, mostly related to the lack of nNOS binding sites and some parts of the central coiled-coil segment of the ROD structure in the utrophin gene [70, 71]. Therefore, utrophin is not fully capable of complementing the function of dystrophin [72], and combinatorial therapies, such as upregulation of utrophin and restoration of dystrophin, can be proposed as an approach with better efficacy. Nevertheless, utrophin alone therapy may be plausible in patients with BMD who still express a low level of dystrophin [73].

The exon skipping strategy with predesigned AONs leads to the conversion of frameshift to in-frame deletions, resulting in the expression of a shortened but functional protein [74]. Typically, AONs are 20–30 nucleotide-long fragments of DNA or RNA, which, by binding to the exon/intron boundary or targeting intraexonic regions, can skip the particular exon(s), ‘hide’ it from the splicing machinery, and restore the reading frame [75]. As this method is a mutation-specific approach (e.g., eteplirsen skips exon 51 of the *DMD* gene, golodirsen and viltolarsen may be used in patients having confirmed exon 53 amenable mutations, while casimersen allows skipping exon 45), different AONs must be used in a form of personalized medicine. Although all four AONs are approved by the FDA [76], several issues regarding their efficacy, applicability, delivery, and cytotoxicity remain questionable [77]. The main drawbacks of AONs include low uptake into heart tissue, with only a temporal effect, and the need for repeated administration, which also affects the cost of the treatment. Nevertheless, multiple exon skipping (multi-exon skipping) or the cocktail of various AONs could theoretically be used to restore the open reading frame of dystrophin mRNA in 80–90% of patients with DMD in total, regardless of the type of mutation [78, 79].

Readthrough therapy can also be applied to only a limited number of patients, who have nonsense mutations in the *DMD* gene (approximately 13% of boys). The mechanism of action of the compounds used in this strategy is based on the ribosomal readthrough of the stop codon on dystrophin mRNA and the expression of functional protein. The exemplary drug, ataluren [80], is conditionally approved in the European Union [2, 81]. Although the main objectives of some clinical trials have not been met, many data indicate that this drug delays the progression of DMD (reviewed in: [82]).

Finally, the application of the CRISPR/Cas9 system, a highly effective genetic editing technology, has great potential to be used for the treatment of DMD. Functional restoration of the dystrophin gene was obtained not only in vitro, in dystrophic myoblasts derived from induced pluripotent stem cells (iPSCs) [83, 84] but also in various animal models of DMD—mice [59, 85, 86], dogs [87], and finally pigs [88]. Although CRISPR/Cas9-mediated dystrophin correction may represent a one-time therapy with long-term results [89], its clinical application is still under debate due to the possibility of off-target gene editing [90, 91], including the risk of unspecific mutations. More details on the latest findings and modifications of this approach could be found in recent reviews [92, 93].

#### Cell therapies

The most controversial and least studied and effective in clinical trials are strategies aimed at the transplantation of cells expressing functional dystrophin. Recently, much attention has been paid to the possible use of meso-angioblasts. These vessel-associated progenitors have been shown to cross the blood-vessel wall after intra-arterial delivery [94] and differentiate into muscle fibers [95]. Despite these valuable abilities, a phase 1/2a clinical study did not elicit a significant benefit [96]. Although other cell populations have also been suggested to be used, including bone marrow-derived mesenchymal stem cells (BM-MSCs) and CD133+ progenitors [97–99], only muscle SCs are proven *bona fide* muscle-derived stem cells able to form new muscle fibers. Unfortunately, the success of their clinical application in humans is so far very limited [100, 101].

#### Pharmacological therapies

Pharmacological compounds, acting through inhibition of NF-κB (corticosteroids–glucocorticoids), downregulation of histone deacetylase activity (HDAC inhibitors), modulation of the utrophin level, or angiotensin activity (angiotensin-converting enzyme inhibitors; ACEIs and angiotensin II receptor blockers; ARB), mostly used as factors mitigating the downstream effects of dystrophin deficiency, have been tested in animal models, as well as clinical trials (reviewed in [4]).

Glucocorticoids: prednisone, prednisolone, and deflazacort have been used as the gold standard for the treatment of DMD for more than 30 years [102, 103]. Their protective effects are mediated mostly through inhibition of NF-κB signaling and reducing the inflammatory response [104, 105]. However, new mechanisms responsible for their beneficial impact have also been identified. St-Pierre et al. [106] observed that in a mouse model, the use of deflazacort leads to increased expression of nuclear factor of activated T cells 1 (*NFATc1*)-dependent genes, including utrophin. However, upregulation of utrophin was not evident in prednisolone-treated myotubes derived from fibroblasts of patients with DMD [107]. Prednisone and deflazacort can activate the expression of genes, such as *Anxa1* and *Anxa6,* which encode proteins involved in the repair of the sarcolemma after injury (annexin A1 and annexin A6, respectively) [108], while Kameyama et al. also found that prednisolone can inhibit matrix metalloproteinase-2 (*MMP-2)* mRNA and consequently increase the level of laminin, the main component of the basement membrane of muscle fibers [107]. All these mechanisms may be responsible not only for the glucocorticoid-triggered reduction of muscle damage, but also for their cardioprotective outcomes [109–111]. Although glucocorticoids delay loss of ambulation in DMD patients, they are accompanied by prominent adverse effects, including excessive weight gain, growth inhibition, adrenal insufficiency, bone weakness, cataract development, and behavioral changes [103]. To reduce some of these negative consequences, the use of new and safer steroid analogs such as vamorolone (also known as VBP15) [112–115] or optimized, less frequent, administration of conventional drugs [108] was suggested. However, a recent comparison of the effectiveness of daily prednisone or deflazacort with intermittent prednisone (10 days on and then 10 days off) found the superiority of the daily corticosteroid regimen over the intermittent treatment [116].

Although the field of pharmacological, gene, and cell therapies for the treatment of DMD continues to advance, new promising therapeutic strategies are being evaluated. An example of such an approach also includes the use of hydrogen sulfide (H_2_S).

## Why H_2_S? How can we increase the level of H_2_S in vitro and in vivo?

Gasotransmitters, small gaseous messenger molecules that are freely permeable to cell membranes, produced endogenously in an enzymatically controlled manner, have profound physiological functions and implications in therapeutics. Their discovery has provided new insights into the mechanisms of signaling and cellular interactions. It turned out that signal transduction can occur without the involvement of membrane receptors: the ligand–receptor mechanism is not the only way to modulate cellular activity, and these roles can also be played by endogenously synthesized gas messengers [117]. The first gasotransmitter identified was nitric oxide (NO), followed by carbon monoxide (CO), and, as the third, H_2_S was classified among gaseous messengers. H_2_S, like CO, has until recently evoked negative associations. This colorless and flammable gas with the characteristic odor of rotten eggs in higher concentrations has adverse effects on the body, including irritation of the eyes and respiratory system, and can even lead to death through inhibition of mitochondrial respiration [118]. However, all three gases have been shown to act as regulators of many biological functions in animals as a consequence of their anti-inflammatory, cytoprotective, antioxidant, and anti-apoptotic properties [119].

**Fig. 3:**
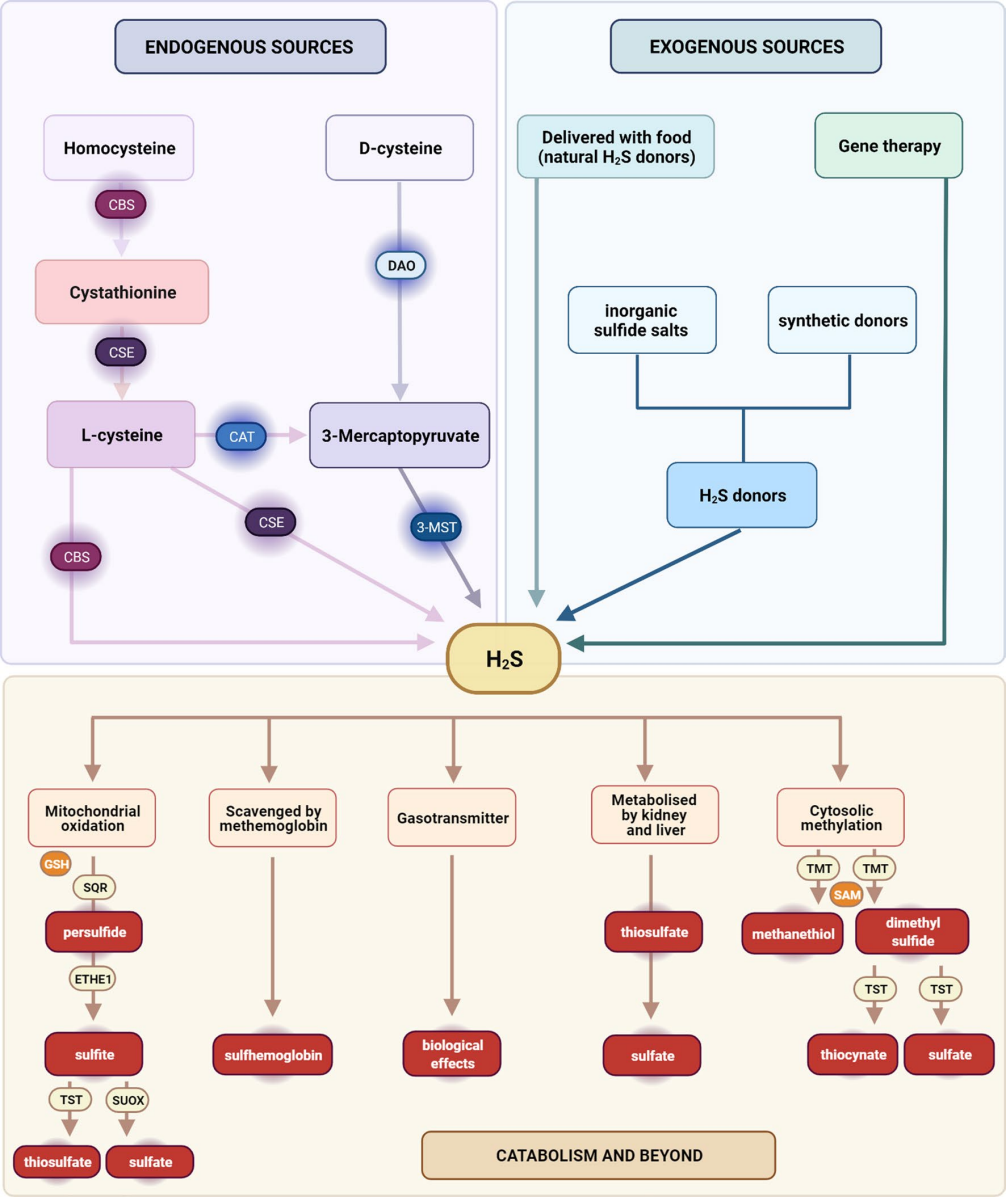
Endogenous and exogenous sources of H_2_S and its further fate. Endogenous synthesis of H_2_S occurs primarily through an enzymatic pathway, and the main precursor is l-cysteine derived from the metabolism of homocysteine and cystathionine. l-cysteine can be converted to H_2_S by cystathionine β-synthase (CBS) or cystathionine γ-lyase (CSE). Other enzymes involved in H_2_S synthesis may be D-amino acid oxidase (DAO) and cysteine aminotransferase (CAT), which convert d-cysteine and l-cysteine, respectively, to 3-mercaptopyruvate, for further metabolization by 3-mercaptopyruvate sulfurtransferase (3-MST). Exogenously, H_2_S can be delivered with food and in the form of chemical donors, including inorganic sulfide salts or new-generation synthetic donors. Another approach to increase the level of gasotransmitter through external sources is gene therapy based on the overexpression of H_2_S-generating enzymes. Subsequently, H_2_S acts as a gasotransmitter, exerting numerous biological effects or is catabolized through several pathways. It can be scavenged by methemoglobin to form sulfhemoglobin, metabolized into thiosulfate and sulfate in the kidney and liver, or exhaled directly by the lungs. The main catabolic pathway is based on oxidation in the mitochondria. In subsequent reactions catalyzed by sulfide quinone oxidoreductase (SQR) and persulfide dioxygenase (ETHE1), H_2_S is converted to a persulfide and sulfite, respectively. Sulfite may be turned into sulfate or thiosulfate by sulfite oxidase (SUOX) and thiosulfate transferase, TST (also called rhodanese). In the cytosol, H_2_S is methylated by thiol *S*-methyltransferase (TMT) to methanethiol and dimethyl sulfide, which can be further processed by TST to thiocyanate and sulfate. GSH, glutathione; SAM, *S*-adenosyl-methionine

In mammals, H_2_S biosynthesis can occur through both enzymatic and non-enzymatic pathways [120] (Fig. 3). Most endogenous H_2_S production is mediated by pyridoxal 5′-phosphate-dependent enzymes: cystathionine β-synthase (CBS), cystathionine γ-lyase (CSE), and 3-mercaptopyruvate sulfurtransferase (3-MST, MPST) using l-cysteine, l-homocysteine or l-methionine as substrates [121]. Although these enzymes can demonstrate organ and tissue-specific distributions, they are all expressed in skeletal muscles, heart, vasculature, and endothelium [122, 123]. An additional biosynthetic pathway was described for the production of H_2_S from d-cysteine, involving 3-MST and d-amino acid oxidase (DAO). However, this mechanism is not universal and is primarily limited to the brain, kidney, and gastrointestinal system [124–126]. Furthermore, Akaike et al. [127] reported that H_2_S can also be produced from l-cysteine by cysteinyl-tRNA synthetases (CARSs) while Pol et al. [128] found selenium-binding protein 1 (SELENBP1), a methanethiol oxidase (MTO), responsible for the production of H_2_S (together with H_2_O_2_ and formaldehyde) from methanethiol. Regardless of the biosynthesis pathway, subsequently, H_2_S acts as a gaseous mediator and can finally be metabolized by oxidation in mitochondria, methylation in the cytosol, or can be scavenged by methemoglobin to form sulfhemoglobin [117, 129] (Fig. 3). Oxidation in mitochondria, which occurs in a two-step reaction, is the major catabolic pathway. First, H_2_S is oxidized by sulfide quinone oxidoreductase (SQR) to a persulfide, which is further oxidized to sulfite by persulfide dioxygenase (ETHE1). Finally, sulfite is converted to sulfate or thiosulfate by sulfite oxidase (SUOX) and thiosulfate transferase, TST (also called rhodanese), respectively. Cytosolic methylation also involves the activity of several enzymes. Thiol S-methyltransferase (TMT) triggers the conversion of H_2_S to methanethiol and dimethyl sulfide, and then, the latter is oxidized to thiocyanate and sulfate due to the activity of TST. The gasotransmitter can also be removed in an unchanged form through exhalation from the lungs. In the kidney, it is metabolized into final products such as thiosulfate and sulfate, while the liver turns H_2_S mainly into sulfate (Fig. 3), (for references, see: [130]).

**Fig. 4:**
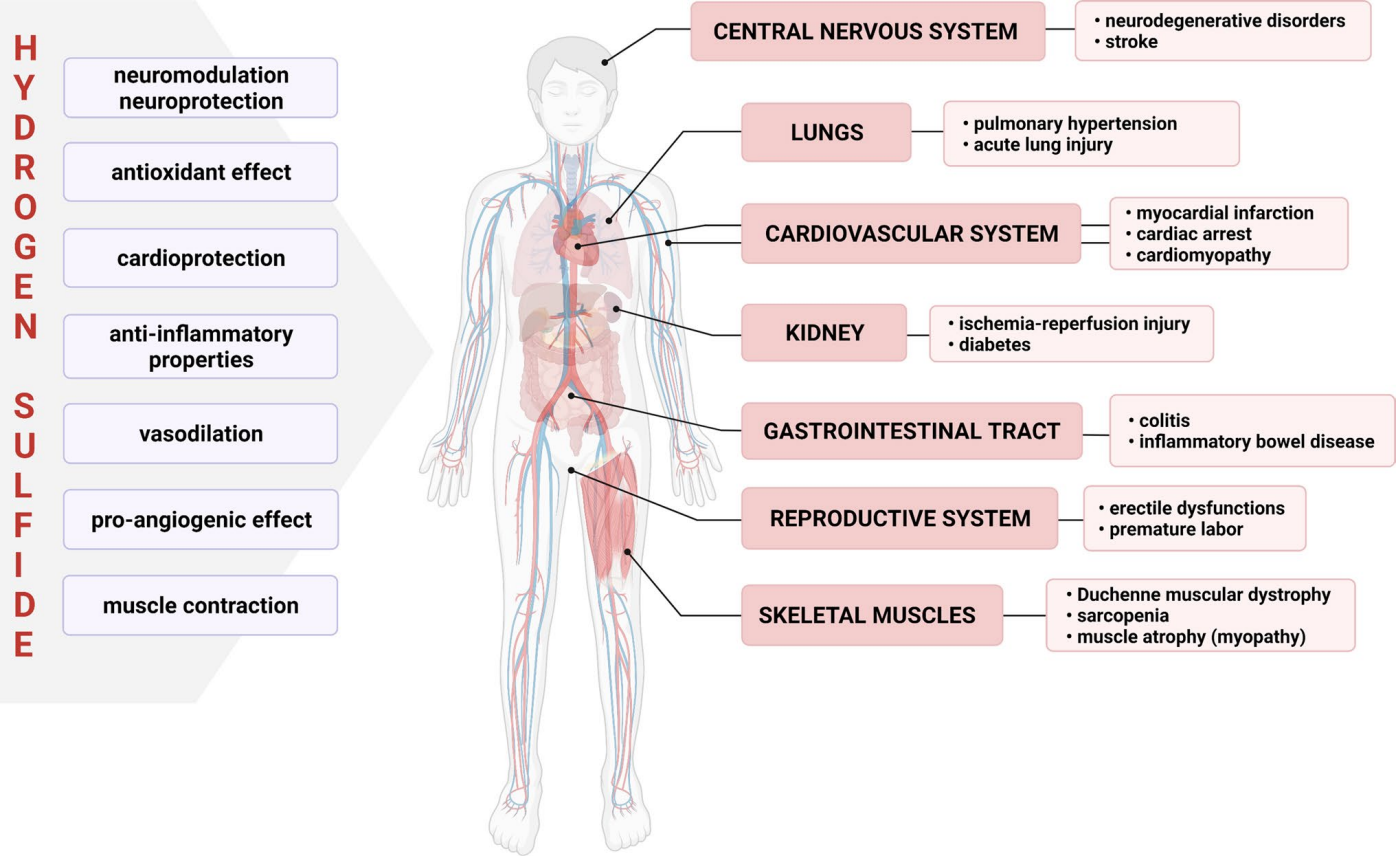
Physiological roles and therapeutic targets of H_2_S. The cytoprotective effects of H_2_S are exerted by the numerous activities that can be demonstrated in the regulation of various organ functions under physiological and pathological conditions. Among others, H_2_S has been proposed as a therapeutic in neurodegenerative disorders, acute lung injury, and myocardial infarction. The protective role of H_2_S has been demonstrated in ischemia–reperfusion injury in the kidney, inflammatory bowel disease and colitis, as well as in reproductive system dysfunctions. Finally, H_2_S may be a preventive factor in skeletal muscle-related disorders, such as muscle atrophy, sarcopenia, and Duchenne muscular dystrophy

H_2_S has been shown to affect different organs and regulate a variety of processes in the organism (Fig. 4). Among others, it acts as a neuromodulator and antioxidant [131], an anti-inflammatory compound [132], a mediator of vasorelaxation [133], a stimulator of angiogenesis [134], a regulator of muscle contractility [135], and a protective factor against heart failure [136]. Furthermore, H_2_S can modify a wide variety of proteins, including those involved in signal transduction pathways, through post-translational modification of protein cysteine residues in a process known as S-sulfhydration (that is, conversion of cysteine -SH groups to -SSH) [137]. Due to this modification, H_2_S activity can be found in virtually all physiological processes, and its possible application to treat neurodegenerative, cardiovascular, renal, and other diseases has been widely investigated [130].

Taking into account the versatile applications of H_2_S, there are attempts to use this mediator as a therapeutic agent. However, it is important to use a physiologically relevant dose of H_2_S to avoid its toxic effect. In rat, human, and bovine brain tissues, H_2_S is present at levels of up to 50–160 μM [138], but there are studies that show its beneficial effects in a wider range of concentrations (10–300 μM) [139]. Several different possibilities of H_2_S delivery are described. The gaseous nature of the compound does not facilitate its direct administration, as its inhalation raises some concerns about the possible toxicity and the problems in estimating the precise dose administered [140]. However, in a mouse model of Parkinson’s disease (induced by the administration of dopaminergic neurotoxin, MPTP), H_2_S inhalation for 8 h/day for 7 days, prevented neuronal apoptosis and protected against disease-induced movement dysfunction [141]. A different research group has shown that H_2_S administered in gaseous form promotes glucose uptake by increasing insulin receptor sensitivity and improves kidney lesions in type II diabetes [142]. For exogenous delivery of H_2_S, donors in the form of sulfide salts or synthetic molecules are often used (Fig. 3, Table 1). The most common inorganic sulfide salts are sodium hydrosulfide (NaSH) and sodium sulfide (Na_2_S). They are water-soluble solid analogs of H_2_S, providing rapid access to biologically relevant forms of sulfide such as H_2_S and HS^−^ [143]. These compounds have been used in many in vitro studies and have broad applications in vivo*.* Importantly, short- and long-term treatment was shown to have a favorable outcome. Chen et al. [144] showed that only a single dose of 50 μmol/kg body-weight NaHS improved cardiac function in the mouse model of sepsis-induced myocardial dysfunction. Also long-term (4 weeks) administration of NaHS to C57BL/6J mice by intraperitoneal injection played a protective role in vascular remodeling and inhibited the inflammatory response by activating PPARδ/SOCS3 signaling pathway [145].

**Table 1:**
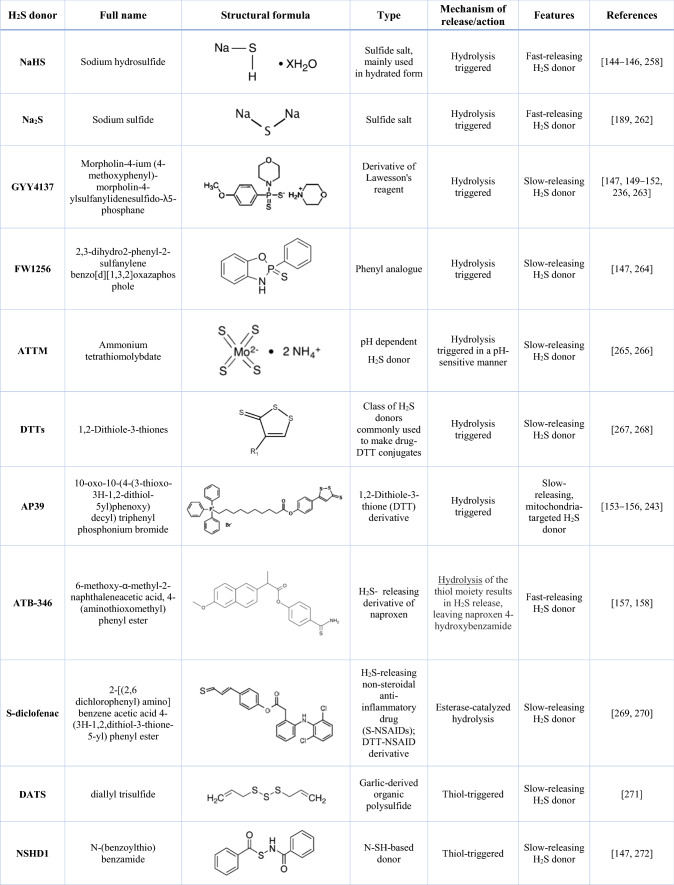

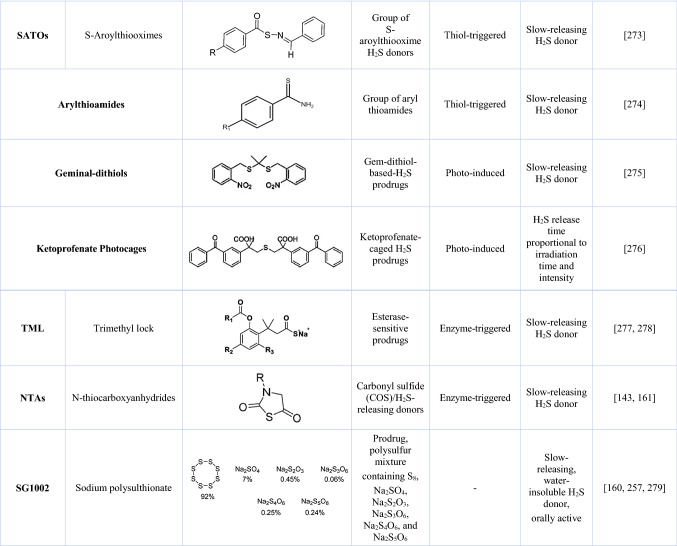
Overview of selected H_2_S donors and their mechanism of action

Although NaHS has been used as the main tool for increasing H_2_S concentration in virtually all disease models for years, its main limitation is related to the very rapid release of the gasotransmitter. Upon reaction with water, NaHS and other sulfide salts hydrolyze immediately, leading to a local and transient supra-physiological H_2_S level followed by a rapid decline [146]. Such poor pharmacokinetic properties limit their use in potential therapies and have led to the discovery of innovative H_2_S donors, such as GYY4137 and FW1256 [147]. GYY4137 releases H_2_S significantly slower (10 min) compared to NaHS (10 s) [148] and is the most studied water-soluble compound that releases H_2_S through hydrolysis. It was used to study the cardioprotective effect of H_2_S in vitro [149] and in vivo [150]. The use of this donor allowed the demonstration that H_2_S can have anti-tumor effects [151], stimulate autophagy, and attenuate ferroptosis [152], among other effects.

AP39 is the example of a mitochondria-targeted H_2_S donor, as it contains a mitochondria-targeting motif, triphenyl phosphonium coupled with an H_2_S-donating moiety (dithiolethione). This compound was shown to protect against oxidative stress-mediated renal epithelial cell injury in vitro and renal ischemia–reperfusion injury in vivo [153]*,* and oxidative mitochondrial DNA damage in endothelial cells [154]*.* Furthermore, in a model of cardiac arrest and cardiopulmonary resuscitation-induced neurological injury, treatment with AP39 preserved mitochondrial integrity, reduced oxidative stress, and improved neurological function and long-term survival rates [155]. The cardioprotective effect was also demonstrated [156].

Among different H_2_S donors, an interesting class of new compounds has been developed that combine traditional nonsteroidal anti‐inflammatory drugs (NSAID) with a chemical moiety that donates H2S. Exemplary compounds, ATB-346, a derivative of naproxen [157, 158] and ATB-352, known as HS-ketoprofen [159], were shown to exert reduced toxic effects in the gastrointestinal track, compared to the parent NSAID. Importantly, their anti-inflammatory and analgesic potential was comparable or even better than traditional NSAIDs. It is noteworthy that the Phase 2 clinical trial (http://ClinicalTrials.gov, NCT03291418) conducted on healthy male and female subjects has demonstrated a superiority of ATB-346 over the naproxen, since less gastroduodenal ulceration was evident in the ATB-346 group [157].

To improve the chemical properties of H_2_S donors and use those molecules in a more controllable way, new derivatives were recently synthesized. Among them, the pH-controlled factor (ammonium tetrathiomolybdate, ATTM) and the thiol-triggered H_2_S releaser (SG1002), after the successful results of phase I clinical trials (http://ClinicalTrials.gov, NCT01989208) [160], are suggested for the treatment of cardiovascular diseases and breast cancer. The field of discovery of new small molecules and materials-based approaches for controlled delivery of H_2_S and related reactive sulfur species (RSS) increases rapidly. Examples of such compounds are N-thiocarboxyanhydrides (NTAs) that release carbonyl sulfide (COS), which is effectively converted to H_2_S by the action of the enzyme carbonic anhydrase (CA) [161]. A summary of basic information on various H_2_S donors is provided in Table 1. However, we encourage the readers to seek more details in other articles that focus on donor chemistry, their pros and cons, and possible applications [140, 162, 163].

**Fig. 5:**
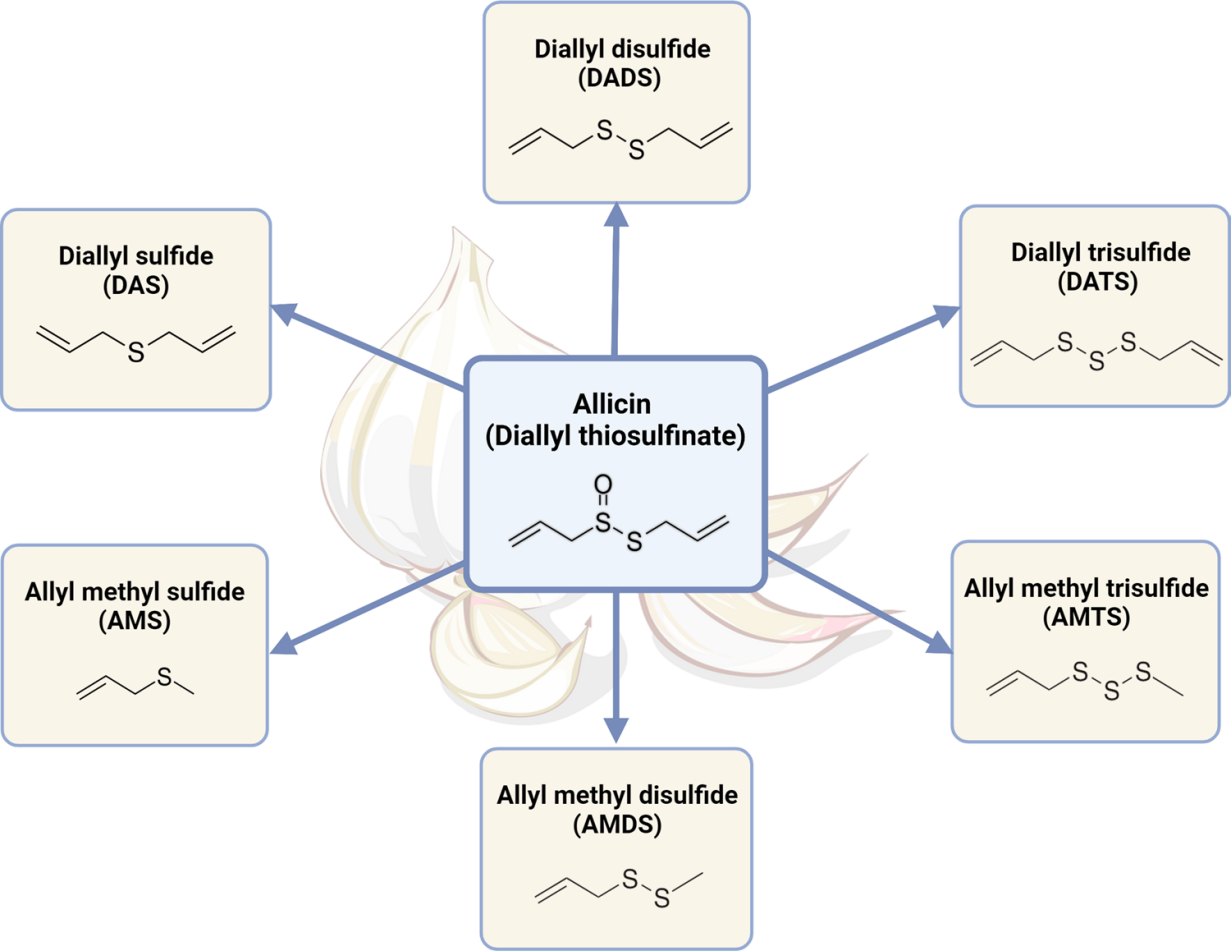
Garlic-derived natural H_2_S donors. Allicin (diallyl thiosulfinate) is an organosulfur, active garlic component. Its decomposition results in the formation of several H_2_S-releasing compounds such as diallyl sulfide (DAS), diallyl disulfide (DADS), diallyl trisulfide (DATS), and methylated forms including allyl methyl sulfide (AMS), allyl methyl disulfide (AMDS), and allyl methyl trisulfide (AMTS). The newly formed compounds are considered natural H_2_S donors with a broad spectrum of biological activity

There are also known natural H_2_S donors, such as garlic extracts (Allium sativum) and derivatives [164]. The most characterized active component of garlic, allicin (diallyl thiosulfinate), unstable in aqueous media, is quickly converted into several H_2_S-releasing compounds [e.g., diallyl sulfide (DAS), diallyl disulfide (DADS), diallyl trisulfide (DATS) and allyl methyl sulfide (AMS), allyl methyl disulfide (AMDS), allyl methyl trisulfide (AMTS)] (Fig. 5). Similarly, isothiocyanates such as sulforaphane and allyl isothiocyanate present in cruciferous vegetables have significant H_2_S-releasing activity. However, the main problem with supplementation with these natural means may be standardization of dosage, as well as bioavailability and absorption of active ingredients.

Finally, another strategy may be based on the gene therapy approach. Overexpression of H_2_S-generating enzymes, leading to increased H_2_S production, may have superior properties over chemical compounds, since the latter may exert functions related not only to H_2_S signaling. Sen et al. [165] have shown that ex vivo gene therapy with plasmids having CBS, CSE, and 3-MST genes improved the relaxation of hyperhomocysteinemic arterial explants. In *D. melanogaster,* constitutive ubiquitous overexpression of CBS extended a lifespan, improved locomotor activity, and increased resistance to hyperthermia (35 °C) [166]. On the other hand, genetic overexpression of CBS in the brain caused dysregulation of serotonin and dopamine pathways [167] and contributed to Down syndrome-associated neuronal disturbances [168].

## Possible mechanisms of beneficial effects of H_2_S

**Fig. 6:**
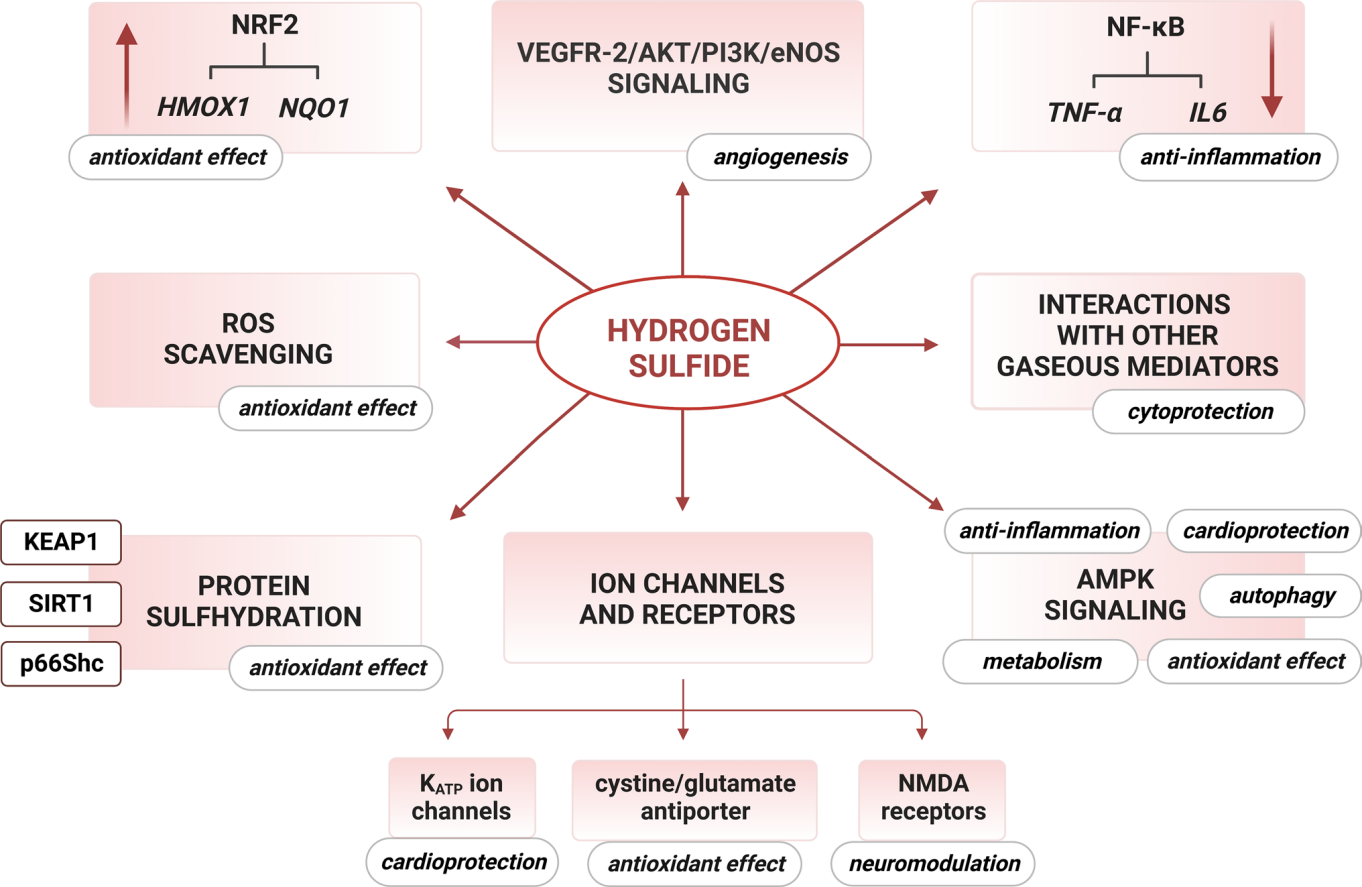
Biochemical functions of H_2_S. Hydrogen sulfide exerts its cytoprotective properties through several mechanisms. Direct quenching of ROS, enhanced cysteine transport that increases GSH, or post-translational S-sulfhydration of, e.g., p66Shc adaptor protein or SIRT1 contributes to an antioxidant effect. The S-sulfhydration of KEAP1 alters its ability to direct NRF2 to ubiquitylation and proteasomal degradation, thus allowing the accumulation of newly translated NRF2, its nuclear translocation, and increased expression of NRF2-regulated downstream genes such as *HMOX1* and *NQO1*. Activation of cardiac K_ATP_ channels mediates the cardioprotective effect of H_2_S. The anti-inflammatory mechanism of H_2_S is related to decreased activation and nuclear translocation of NF-κB, resulting in reduced transcription of pro-inflammatory genes, such as *TNF-α* or *IL-6*. Furthermore, H_2_S-mediated cytoprotection can involve activation of AMPK. AMPK also mediates antioxidant and anti-inflammatory effects and contributes to the regulation of autophagy and metabolism. Another mechanism of H_2_S action is related to its pro-angiogenic activities and may involve VEGFR/PI3K/AKT/eNOS signaling. Neuromodulatory functions are achieved by activating N-methyl-D-aspartate (NMDA) receptors, among other mechanisms. The interaction with NO and CO also contributes to the cytoprotective effect

H_2_S exerts anti-inflammatory, anti-apoptotic, and antioxidant activities (Fig. 6). Other known effects are related to neuroprotection (e.g., by mediating the *N*-methyl-d-aspartate, NMDA) receptor responses) and relaxation of blood vessels (e.g., by enhancing the effects of NO), lowering blood pressure, and regulation of angiogenesis by affecting multiple pathways and interactions with ion channels, enzymes, transcription factors, and receptors [169, 170]. H_2_S may, similarly to NO, upregulate the activity of soluble guanyl cyclase (sGC) and increase the level of cGMP. This could be at least in part due to direct inhibition of cGMP phosphodiesterase (PDE5) activity, leading to an increase in the half-life of cGMP [170]. As oxidative stress, inflammation, and angiogenesis, disturbances greatly contribute to the progression of DMD and the leading cause of death is related to dilated cardiomyopathy and other cardiac dysfunctions, below we focus on these aspects of H_2_S-mediated cytoprotection.

### H_2_S as an antioxidant

Oxidative stress is caused by the overproduction of ROS relative to cellular antioxidant defense. H_2_S exerts antioxidant properties through several mechanisms, including direct quenching of ROS [171] and reactive nitrogen species (RNS) [172], increasing antioxidant levels, and regulating the state of certain proteins through S-sulfhydration [137].

An essential pathway associated with maintaining redox balance by H_2_S is the activation of the nuclear factor-erythroid 2-related factor 2 (NRF2; encoded by the *Nfe2l2* gene), which regulates the expression of numerous antioxidant genes [173, 174]. Under normal conditions, NRF2 is located in the cytoplasm and is bound to its negative regulator, the Kelch-like ECH-associated protein 1 (KEAP1) dimer, which targets NRF2 for proteasomal degradation in a ubiquitin-dependent manner. H_2_S interacts with and modifies KEAP1 through S-sulfhydration, resulting in its conformational change and preventing ubiquitylation of bound NRF2. Newly synthesized NRF2 is not sequestered by KEAP1, but can accumulate in the nucleus, where it binds to the antioxidant response element (ARE) and leads to induction of the expression of NRF2-regulated downstream genes [175–177]. One of them is heme oxygenase-1 (HO-1; *HMOX1*/*Hmox1*), which exerts anti-inflammatory and antioxidant properties. HO-1 plays a protective role in many pathological conditions, including the cardiovascular, renal, and central nervous systems [178–180]. Recently, its role in skeletal muscle differentiation and repair [181–183] and the progression of DMD [184] has also been revealed. Other NRF2-regulated genes encode proteins that are strongly involved in the mitigation of oxidative stress with ROS scavenging, such as thioredoxin (TRX), glutathione S-transferase (GST), glutathione peroxidase (GPx), thioredoxin reductase (TRXR), and catalase [185]. The involvement of NRF2 and its downstream effectors in the antioxidative response of H_2_S has been demonstrated in many studies. For example, the NRF2/HO-1 signaling pathway was activated by NaHS treatment in a rat model of cyclophosphamide-induced toxicity [186]. It should be noted that simultaneously with NRF2 induction, the level of NRF2 targets, such as NAD(P) H quinone oxidoreductase 1 (NQO1), reduced glutathione (GSH), and superoxide dismutase (SOD), also increased. HO-1 was induced by H_2_S and has cardioprotective effects in rats with volume overload-induced heart failure [187], and in mice with coxsackievirus B3-induced myocarditis [188]. Calvert et al. [189] demonstrated induction of NRF2, HO-1, and Trx-1 in ischemic cardiac tissue after Na_2_S treatment, leading to a reduction in infarct size, the level of cardiac injury marker, troponin I, and oxidative stress. The protective outcome of H_2_S-induced HO-1 expression was also shown in pulmonary hypertension [190] and wound healing [191].

Modulation of GSH concentration is another key mechanism in the antioxidant effect of H_2_S. GSH, a tripeptide, γ-l-glutamyl-l-cysteinylglycine, is synthesized in consecutive reactions catalyzed by two enzymes, glutamate-cysteine ligase (GCL) and glutathione synthetase (GS). Kimura et al. [171] demonstrated that H_2_S enhances the transport of the sulfur amino acid precursor, cysteine, to increase GSH production. Furthermore, upregulation of the expression of GCL subunits: a catalytic subunit (GCLC) and a modifier subunit (GCLM) by H_2_S leads to increased intracellular GSH levels [192].

The antioxidant functions of H_2_S may also involve the regulation of AMPK. The reduction of oxidative stress and apoptosis by NaHS in an experimental model of aging was mediated by activation of the calmodulin-activated protein kinase kinase-β (CaMKKβ)/AMPK pathway [193]. A similar cytoprotective effect of NaHS through activating AMPK was found in a model of dexamethasone-induced osteoblast cell damage [194]. Furthermore, ajoene, a garlic by-product, demonstrated antioxidant properties in hepatic steatosis induced by a high-fat diet (HFD) through stimulation of AMPK signaling [195].

As mentioned above, S-sulfhydration is an important mechanism of H_2_S-mediated signaling. This post-translational modification of the p66Shc adaptor protein greatly contributes to the antioxidant effect exerted by H_2_S. p66Shc is involved in mitochondrial redox signaling and its phosphorylation at Ser36 leads to increased ROS production by three different mechanisms in a manner bound to the mitochondria, the plasma membrane, and the nucleus [196, 197]. Oxidative stress associated with p66Shc activity is an important factor in the pathology of many diseases and pathological conditions, including DMD [198], diabetes mellitus, hypercholesterolemia, endothelial dysfunction, and aging (reviewed in [197]). Xie et al. [199] revealed that H_2_S persulfidates p66Shc at Cys59, which is located near Ser36, preventing the phosphorylation of p66Shc, its translocation to mitochondria, and therefore inhibiting ROS production. These effects were achieved by NaHS treatment and CBS overexpression, which means that exogenous and endogenous H_2_S reduces oxidative stress. Interestingly, p66Shc is negatively regulated by sirtuin1 (SIRT1) [200]. SIRT1 belongs to the family of nicotine adenine dinucleotide (NAD+)-dependent histone deacetylase proteins, enzymes that catalyze post-translational modifications of many proteins [201]. SIRT1 acts on multiple targets, such as p53, FOXO3, and eNOS, and exhibits diverse biological activities, including negative regulation of oxidative stress (antioxidant properties) [197]. Notably, SIRT1 was suggested to provide beneficial effects in mouse models of DMD through the attenuation of oxidative stress, inflammation, and fibrosis [202]. Increased SIRT1 activity can be induced by direct S-sulfhydration by H_2_S [203], implying that some of the antioxidant properties of the gasotransmitter can be mediated by regulation of histone deacetylase activity. It is supported by studies in which in vitro application of H_2_S decreased endoplasmic reticulum stress [204], oxidative stress and senescence [205], while application of SIRT1 inhibitors reversed these effects.

## Cardioprotective roles of H_2_S

The synthesis of endogenous H_2_S in the heart occurs predominantly through the activity of CSE and substantially less by CBS [206, 207] and the CSE/H_2_S pathway was shown to be particularly important for the proper functioning of the cardiovascular system. Cardioprotective effects of H_2_S can result from multiple mechanisms of action, including reduction of oxidative stress, anti-apoptotic and anti-inflammatory effects. Among various possible molecular pathways, H_2_S-mediated cardioprotection may involve AMPK activation, which was demonstrated in cardiac arrest in mice [208], myocardial ischemia/reperfusion injury [209], cigarette smoking-induced left ventricular dysfunction in rats [210], and high-fat diet-induced diabetic cardiomyopathy [211]. Nevertheless, the influence on ion channels that affect cardiac contractility appears to be the main cardioprotective mechanism of H_2_S.

ATP-sensitive potassium (K_ATP_) channels are widely distributed and can be found in the heart, muscles, pancreas, and brain [212, 213]. Being highly sensitive to ATP and ADP concentrations, the channels are high-fidelity sensors that match membrane excitability with a given metabolic state of the cell [214]. K_ATP_ activation prevents uncontrolled calcium influx, stabilizes membrane potential, and regulates cardiac contractility [215]. Disruption of K_ATP_ channel functions has been associated with the progression of many diseases and conditions, including hypertension [216], ischemic heart disease [217], dilated cardiomyopathy [218], or cardiomyopathy in DMD [219]. In myopathic hearts, these channels exhibit abnormal responses to the metabolic state of the cell, particularly to ATP and CK concentrations, resulting in impaired regulation of membrane excitability, which exposes cardiomyocytes to calcium loading and necrosis [220]. Some of these alterations can be mitigated by compounds known to open K_ATP_ channels, and interestingly, one of them is H_2_S [221]. The channels are built from pore-forming subunits, Kir6.x (Kir6.1 or Kir6.2) and a sulfonylurea receptor (SUR1, SUR2, SUR2A, SUR2B), having regulatory activity. H_2_S was shown to trigger the opening of SUR1 in vascular smooth muscle cells [221] and SUR2B in colonic smooth muscle cells by S-sulfhydration [222]. Mys et al. [223] reported a simultaneous increase in SUR2 and Kir6.1 mRNA levels along with CSE and 3-MST in rat hearts treated with pyridoxal-5-phosphate (PLP) as a cofactor of enzymes that synthesize H_2_S. This implies that the S-sulfhydration process is also involved in the activation of cardiac channels. The cardioprotective effect of H_2_S may result from acting through sarcolemmal K_ATP_ channels and/or mitochondrial K_ATP_ channels. Bian et al. [207] demonstrated the cardioprotective effect of H_2_S by opening sarcolemmal K_ATP_ channels. Increased cell viability and decreased severity and duration of arrhythmias after ischemia/reperfusion in cardiac myocytes treated with NaHS were reported. Blocking sarcolemmal K_ATP_ channels reversed the beneficial effects of the H_2_S donor, whereas inhibiting mitochondrial K_ATP_ did not cause any change. On the other hand, Liang et al. [224] showed that the cardioprotective effect of H_2_S, manifested by increased cell viability, decreased number of apoptotic cells, and reduced oxidative stress, is abolished after inhibition of mitochondrial K_ATP_. Similarly, Testai et al. [225] reported that beneficial effects of the H_2_S donor in the post-ischemic recovery of the myocardium were antagonized by a mitochondrial K_ATP_ channel blocker. Despite such diverse data on the involvement of sarcolemmal and/or mitochondrial K_ATP_ channels, H_2_S-mediated cardioprotective effects are well-documented.

### Anti-inflammatory and anti-fibrotic effects of H_2_S

The cardioprotective properties of H_2_S may also be related to its anti-inflammatory effect. The inflammatory response induced by tissue damage results in the recruitment of immune cells, followed by a remodeling phase with fibrosis events. Long-term inflammation and defects in the regulatory feedback of inflammatory mediators can facilitate increased deposition of collagen and other matrix proteins, leading to fibrosis and induction of cardiac ventricular dilatation [226]. An important anti-inflammatory mechanism of H_2_S is related to inhibition of activation and nuclear translocation of NF-κB [227, 228], resulting in reduced transcription of pro-inflammatory genes, such as TNF-α or IL-6. NF-κB is a central mediator of inflammation and immune processes, which was found to be chronically active in many diseases with prolonged inflammation, including DMD [229]. Interestingly, Zhang et al. [230] reported that the reduced recruitment of CD11b^+^Gr-1^+^ myeloid cells into the myocardium may be behind one of the mechanisms of the anti-inflammatory effects of H_2_S with particular cardioprotective importance. Another mechanism involves IL-10/JAK/STAT-3-dependent signaling [231]. Finally, inhibition of inflammation may also be dependent on activation of AMPK [232].

H_2_S plays a physiological role in the prevention of fibrosis development. This aspect was broadly evaluated in different organs, for example, the kidney. CSE deficiency resulting in decreased H_2_S and GSH levels caused renal fibrosis with tubular damage, infiltration of inflammatory cells, and deposition of ECM components [233]. On the other hand, exogenous H_2_S was shown to inhibit the expression of fibrotic cytokines and other mediators and the activation of myofibroblasts that leads to the suppression of renal fibrosis [234]. Other studies evaluated the impact of H_2_S on cardiac fibrosis. Exogenous delivery of H_2_S in a form of *S*-propargyl-cysteine in a special liposomal formulation, which leads to the slow release of the active gasotransmitter, had cardioprotective and anti-fibrotic effects by inhibiting the TGF-β1/SMAD signaling pathway [235]. The same signaling pathway was attenuated by treating spontaneously hypertensive rats with another H_2_S donor, GYY4137, leading to inhibition of myocardial infarction [236].

### Modulation of angiogenesis by H_2_S

Another protective effect of H_2_S is related to its pro-angiogenic activities. NaHS treatment increased endothelial cell migration, proliferation, and tube formation in Matrigel in an Akt-dependent way, as well as promoted neovascularization in vivo in the Matrigel plug assay in mice [237]. Similarly, Papapetropoulos et al. demonstrated delayed wound healing and neovascularization in CSE^−/−^ mice, while direct administration of H_2_S donor to injured skin stimulated wound closure in a rat model. Concomitantly, in vitro studies (using endothelial cells) and ex vivo experiments (done on aortic rings isolated from CSE-deficient mice) underscored the cross talk between H_2_S synthesis and the pro-angiogenic action of vascular endothelial growth factor (VEGF) [238]. Recent data reveal more details on the molecular mechanisms responsible for the H_2_S-dependent promotion of angiogenesis. Among them, the involvement of specific microRNAs, for example, upregulation of miR-192 [239] or miR-126-3p [240] has been suggested.

## H_2_S in healthy and diseased skeletal muscles

Initially, mouse skeletal muscles express were shown to express very low levels of H_2_S-generating enzymes or completely lack these proteins [241]; however, Lu et al. [242] and Ellwood et al. [243] were able to detect the CSE protein by Western blotting in mouse gastrocnemius muscle. Discrepant data were also shown when the level of the third enzyme, 3-MST, was analyzed. Its protein level was very low or even undetectable in mouse skeletal muscles [244], while strong expression has been shown in gastrocnemius muscle of C57BL/10ScSn mice [243]. Zhang et al. [245] found that although CSE, CBS, and 3-MST mRNA were expressed in mouse skeletal muscle, the level of CBS protein was negligible. Although in the work by Zhang et al. [245], both CSE and 3-MST were easily detected at the protein level, additional analysis (for example, with the use of DL-propargylglycine (PPG), a CSE-specific inhibitor) revealed that CSE is the main enzyme in mouse skeletal muscles. In rats, detectable amounts of all three enzymes were evident [246], while in human skeletal muscles, high expression of CBS and CSE has been demonstrated [241]. These variations might be related to several divergences in the applied methodology, e.g., concentration and type of antibodies, but they may also indicate species-dependent regulation. However, an increase in H_2_S content (by pharmacological treatment or gene delivery of H_2_S-generating enzymes) may have plausible effects on skeletal muscle biology in various models.

The level of H_2_S and H_2_S-generating enzymes declines with age and during various pathological conditions. In the tibialis muscles of 51-week-old mice, CSE expression and H_2_S production decreased compared to 10-week-old animals. The level of cysteine, the main substrate for H_2_S production, was also reduced in the muscles of elderly mice [245]. A lower endogenous H_2_S concentration was detected with a concomitant reduction in NRF2 cytoprotective factor and a significantly increased oxidative stress in the skeletal muscles of patients with critical limb ischemia [247]. H_2_S levels in serum and muscles and the expression of H_2_S-generating enzymes were decreased in diabetic mice [242] and rats [248] compared to control animals.

### Skeletal muscle-protective effects of H_2_S

Taking into account the diversity of protective effects exerted by H_2_S, it is surprising that its role in skeletal muscles and various muscle diseases has not been extensively evaluated. However, recent experiments indicated that H_2_S can play a role in the regulation of muscle health [123]. Some assumptions on the possible role of H_2_S in skeletal muscles could be analyzed based on the results of studies in patients with homocystinuria. The decrease in CBS expression/activity in these patients resulted in morphological abnormalities in many organs, including muscles, and represented by fragmented Z disks and disorganized myofilaments with collagen deposits in the basal lamina [249]. Some indications of the importance of H_2_S in muscles also come from animal models lacking the activity of H_2_S-generating enzymes. CTH-deficient (Cth^−/−^) mice on a low cysteine diet were characterized by reduced glutathione levels in skeletal muscles and higher levels of autophagy regulators, LC3 and p62, in skeletal myofibers. Enhanced autophagy was related to acute skeletal muscle atrophy (myopathy) and resulted in severe paralysis of the extremities [250].

Only a few studies describe the molecular mechanism of H_2_S activity in healthy and diseased skeletal muscles. Bitar et al. [248] concentrated on the evaluation of NaHS treatment on the development of sarcopenia. Such loss of skeletal muscle mass and impaired functions has been described as a complication in diabetic patients; therefore, in this study, Goto Kakizaki (GK) rats (model for type-2 diabetes) with decreased systemic and muscle H_2_S bioavailability were used. Increased muscle mass and strength and decreased myostatin levels were evident in NaHS-treated diabetic animals compared to controls. In GK rats, an increase in ROS generation was evident, but NaHS delivery resulted in a lower level of superoxide and hydrogen peroxide (H_2_O_2_) in the muscle membrane and mitochondrial fractions, as well as better antioxidant capacity measured by the GSH/GSSG ratio [248]. Similar findings were found in vitro in the mouse C2C12 myoblast cell line. After stimulation with NaHS, an increase in GSH level and a reduction in ROS generation were observed [251]. On the other hand, the knockdown of CSE with siRNA resulted in the opposite effects, with increased H_2_O_2_ generation and decreased expression of enzymes in the GSH biosynthesis pathway. This indicated that H_2_S is an important modulator of oxidative balance in myoblasts and skeletal muscles.

Another work, performed in diabetic mice, showed that H_2_S exerts muscle-protective effects through S-sulfhydration of the muscle RING finger 1 (MuRF1) [242]. MuRF1 (also known as TRIM63) is an E3 ubiquitin ligase, and its increased expression is responsible for muscle mass loss in diabetic conditions. NaHS treatment of db/db mice attenuated skeletal muscle mass atrophy, decreased ROS production, and reduced the degradation of myomesin-1 (MYOM1) and myosin heavy chain 4 (MYH4). Analysis performed in vitro in the C2C12 myoblast cell line revealed that H_2_S modified MuRF1 by S-sulfhydration at Cys44. This mechanism was suggested to be responsible for the reduction in MYOM1 and MYH4 ubiquitination leading to the attenuation of skeletal muscle atrophy [242].

Zhang et al. [245] performed an interesting study on the evaluation of H_2_S effect on myogenesis and concluded that this gaseous molecule is capable of inducing regeneration of skeletal muscle. Cardiotoxin-induced injury, as well as age-dependent sarcopenia, were accelerated under conditions of CSE deficiency, while NaHS treatment promoted myogenesis. The mechanism behind this effect was related to the facilitation of heterodimer formation between myogenic factors: myocyte enhancer factor 2 (MEF2c) and myogenic regulatory factor-4 (MRF4) and the promotion of their binding to the myogenin promoter. Therefore, an increase in H_2_S level might be suggested as a possible treatment of muscle injuries or/and as a preventive factor for age-related sarcopenia.

H_2_S may also act as an anti-fibrotic agent. Attenuated skeletal muscle fibrosis (decreased mRNA levels of *Col1a1*, *Col1a3,* and *TGFb*, as well as collagen content measured by trichrome staining, and regulation of MMPs) was evident in the mouse contusion model. Furthermore, reduced inflammation (lower levels of pro-inflammatory cytokines and chemokines) and oxidative stress (decreased expression level of a key subunit of NADPH oxidases, *gp91phox*) were also present in the gastrocnemius muscles [252]. Although this study shows the pleiotropic effect of H_2_S in reducing skeletal muscle injury, the conclusions are mainly based on the assessment of the level of mRNA and should therefore be interpreted with caution. Nevertheless, the protective effect of H_2_S against fibrosis was also found in diabetic diaphragms [253]. In the rat model of streptozotocin-induced diabetes, NaHS supplementation reduced collagen deposition, slightly decreased pro-fibrotic *Col1a1*, *Col1a3,* and *TGFb* levels, as well as pro-inflammatory cytokines (IL-1β, IL-6, IL-18, and TNF-α), and finally, suppressed activation of the NLRP3 inflammasome. These effects contributed to the better biomechanical properties of the diaphragm [253].

Another important aspect of the protective effect of H_2_S on the diaphragm was demonstrated in the ventilator-induced diaphragm dysfunction (VIDD) model [254]. VIDD is a common problem in patients who undergo mechanical ventilation (MV) for the treatment of hypoventilation. The main complications: the decrease in contractile properties of the diaphragm and oxidative stress, which contribute to diaphragm failure, were eliminated by H_2_S. The H_2_S donor protected against VIDD by abrogating mitochondrial dysfunction and activation of calpain and caspase-3 protease in diaphragm fibers [254].

Finally, the requirement of H_2_S for proper muscle vascularization has been underlined [255]. Intramuscular injections with adenoviral vectors-expressing mouse CSE led to higher muscle H_2_S production and improved muscle functions. The number of CD31-positive blood vessels increased in the gastrocnemius muscle after CSE overexpression, indicating improved vascular density. This study validated CSE as a necessary factor for VEGF-mediated neovascularization in vivo. Also in CBS^±^ mice subjected to hind-limb femoral artery ligation (FAL), treatment with a long-acting H_2_S donor, GYY4137, resulted in a pro-angiogenic effect and improved muscle-tissue vascularization and blood-vessel function, The H_2_S donor was also able to counteract the post-FAL-triggered reduction in blood flow and collateral vessel density [256]. Additionally, the downregulation of pro-angiogenic HIF1-α, VEGF, PPAR-γ, and phosphorylated-eNOS was reversed in animals treated with H_2_S donor. Together, this indicates that H_2_S can mitigate neoangiogenic defects in skeletal muscles.

### The possible roles of H_2_S in DMD

Hypothetically, many DMD-related complications could potentially be alleviated by H_2_S due to known mechanisms of gasotransmitter activity. However, until recently, the scientific literature has not broadly addressed the role of H_2_S in DMD.

The initial study focused on evaluating the cardioprotective potential of H_2_S. A brief scientific report (published in the form of the conference abstract) by Cain et al. [257] shows that supplementation of ‘humanized’ dystrophic mice (*mdx*^*4cv*^*/mTR*^*G2*^ mouse model with ‘humanized’ telomere lengths) with an orally active slow-release H_2_S prodrug (SG1002) results in maintenance of the ejection fraction (ET) at a level similar to wild-type mice, indicating preserved cardiac function. Furthermore, a decreasing trend in cardiac fibrosis was observed in the treated animals *versus* the untreated group. Although these findings need to be confirmed and expanded, we can hypothesize that H_2_S may support the functionality of dystrophic cardiomyocytes and may be a possible therapeutic option to improve DMD-related cardiomyopathy.

In addition to cardioprotective effects, Cain et al. found attenuated fibrosis in the gastrocnemius and diaphragm after SG1002 treatment [257]. More studies demonstrating the beneficial muscle-related effects of H_2_S in mitigating the progression of DMD were published in 2021. When the expression of enzymes related to H_2_S production (CBS, CSE, 3-MST) was analyzed, a significantly decreased level was detected in *mdx* mice, as well as in human primary myoblasts isolated from DMD donors [258]. Although Ellwood et al. did not observe such a prominent effect in *mdx* mice, they demonstrated a potent decrease in total sulfide and 3-MST and CSE levels in dystrophin/utrophin double knockout mice (representing a more severe model of DMD than *mdx* mice) [243]. Additionally, in *mdx* animals, a reduction in the levels of metabolites associated with the transsulfuration pathway (TSP) such as glycine, glutathione, methionine, glutamate, and taurine was evident [258]. Interestingly, changes were observed in young animals and became more severe with disease progression. Furthermore, it follows that *mdx* mice exhibit physiological similarities to animal models of cystathioninemia/cystathioninuria (CSE^−/−^ mice), including reduced expression of a major cellular antioxidant, GSH, in skeletal muscles. NaHS treatment, both short-term (2 weeks) and long-term (12 weeks), had a prominent effect in reducing fibrosis, necrosis, and inflammatory-cell infiltration. Furthermore, reactivation of the autophagy process was also evident after H_2_S delivery. In the quadriceps muscle of *mdx* mice, normalization of decreased levels of autophagy regulators (*Atg3*, *Atg7*, *Atg12*, and *Ulk1)*, elevated expression of pro-inflammatory cytokines (*Il1β*, *Il6*, *Tnfα)*, and pro-fibrotic *Tgfb* was demonstrated after NaHS. Finally, the effect of H_2_S on preventing loss of locomotor activity was shown in *mdx* mice subjected to the rotarod and weight test [258].

Saclier et al. [259] investigated the role of H_2_S in chronic inflammation in DMD. Using a mouse model (*DMD*^*mdx4Cv*^), they demonstrated that dystrophin-deficient myofibers stimulate a transition of macrophages to a pro-inflammatory phenotype, which is equivalent to induction of muscle fibrosis. NaHS inhibited the development of inflammation, as reflected by a decrease in the number of macrophages expressing pro-inflammatory markers (TNF-α, iNOS), and an increasing percentage of macrophages positive for the anti-inflammatory marker CD206. The mechanism of suppression of inflammation may involve the phosphorylation of the AMPK catalytic α1 subunit, AMPKα1 what leads to the acquisition of the macrophage anti-inflammatory phenotype. Unlike the potent regulation of inflammation, treatment of dystrophic mice with NaHS for 3 weeks caused only a very moderate (approximately 10%) decrease in collagen accumulation in the tibialis anterior, while it did not attenuate fibrosis in the diaphragm [259].

Not only were mouse models applied to check whether H_2_S donors may have therapeutic potential in DMD. In the *C. elegans* DMD model with a nonsense mutation at position 3,287 of the DYS-1 dystrophin ortholog, supplementation with H_2_S donors improved motor skills, contractile force, and muscle mitochondrial structure [243]. Although the overall lifespan of mutated worms was not affected by H_2_S supplementation, the delay in muscle cell death was evident, suggesting an improvement in healthspan. An interesting observation was found when two different H_2_S donors were compared: a slow-release sodium GYY4137 (NaGYY) was effective at a considerably higher dose (100 μM) than the mitochondria-targeted donor—AP39 (100 pM). It should be noted that the positive effect with AP39 indicates that the direct action of H_2_S in the mitochondria is sufficient to exert DMD protective effects. In addition, similar results were obtained when *C. elegans* was treated with prednisone, a corticosteroid drug approved for use in patients with DMD.

**Fig. 7:**
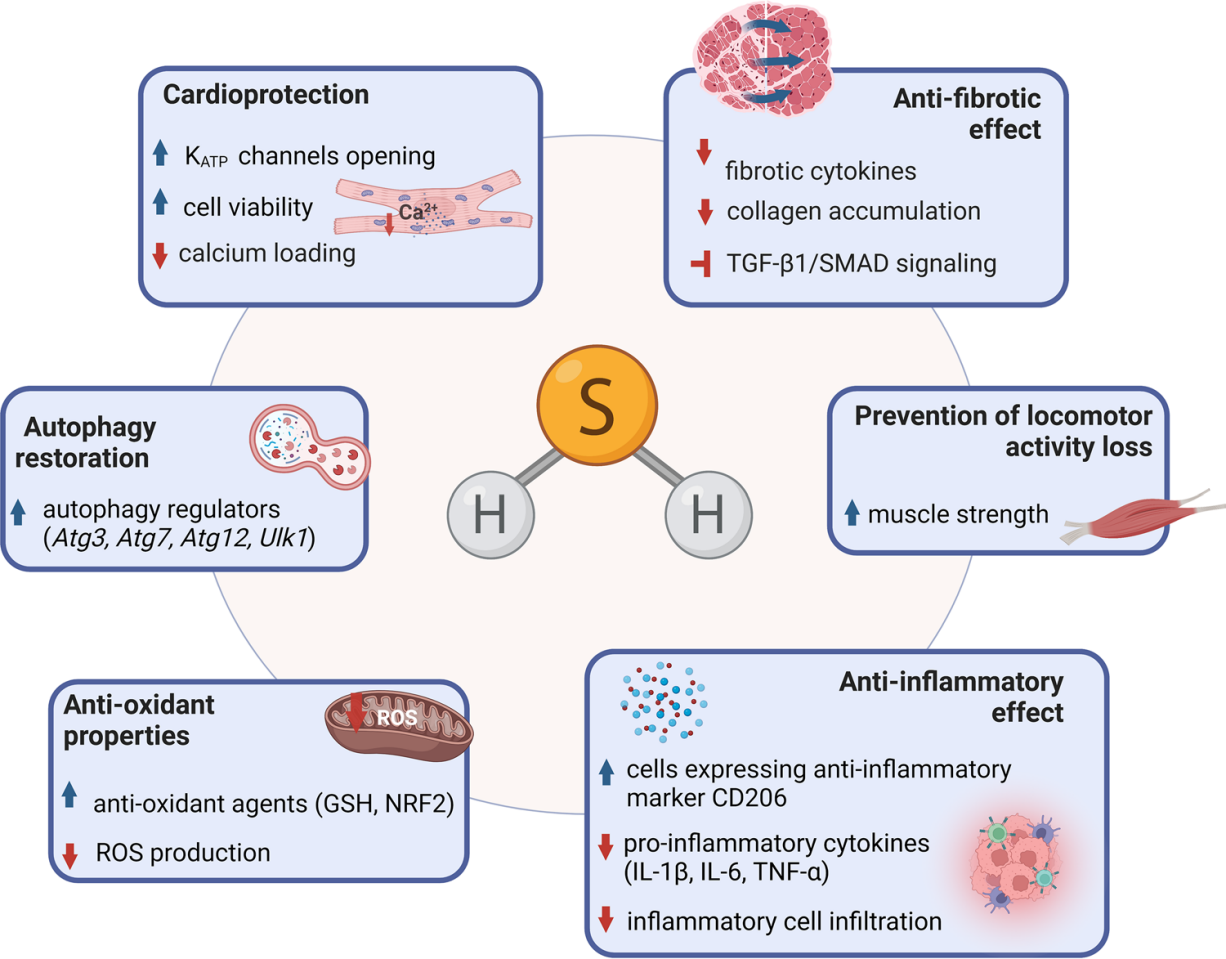
Potential roles of H_2_S in counteracting DMD. Several mechanisms of the cytoprotective effect of H_2_S on the dystrophic phenotype are suggested. The anti-fibrotic effect is the result of reduced fibrosis-related cytokine production, decreased collagen accumulation, the main component of the ECM, and inhibition of the TGF-β1/SMAD signaling pathway. H_2_S prevents loss of locomotor activity by enhancing muscle strength. The improvement in the DMD phenotype is also caused by the anti-inflammatory properties of H_2_S, as indicated not only by an increase in the number of cells expressing anti-inflammatory markers such as CD206, but also by a decrease in the production of pro-inflammatory cytokines and infiltration of inflammatory cells. H_2_S increases the expression of autophagy regulators and antioxidant enzymes while simultaneously preventing ROS production. Furthermore, it exerts a cardioprotective effect by opening K_ATP_ channels, thus reducing calcium loading and preserving cell viability

Although data on the influence of H_2_S donors on the progression of DMD are limited, initial published results strongly support the hypothesis that this gasotransmitter can delay disease progression, by attenuating inflammation and fibrosis and improving muscle strength. Future studies should carefully consider the dose and route of administration of various H_2_S donors, as well as analyze different DMD models. Despite the fact that already published studies utilized discrepant protocols in terms of dose and duration of treatment with H_2_S donors, as well as their route of administration and animal age, they suggest that H_2_S is a beneficial factor in mitigating the dystrophic phenotype (Fig. 7).

## Limitations and future directions

Despite enormous work in the field of the discovery of new therapeutic strategies as well as optimization of the currently available possibilities to treat DMD, the disease is incurable. Although muscle wasting is one of the main hallmarks of DMD, it is not only a skeletal muscle disorder. Therefore, the ideal drug/approach should regulate many symptoms and be able to deal with all the harmful consequences of dystrophin deficiency in other organs, including the diaphragm and heart.

H_2_S, due to its anti-inflammatory, antioxidant, anti-fibrotic, pro-angiogenic, and cardioprotective properties, seems to be an attractive candidate. As mentioned above, this gasotransmitter affects many signaling pathways, including AMPK activity. AMPK serves as a metabolic sensor and regulates lipid and glucose metabolism, and metabolic alterations contribute to the progression of DMD [260] and were also recapitulated in *mdx* mice [261]. Therefore, H_2_S, in addition to alleviating muscle-related symptoms of DMD, can hypothetically affect dysregulated metabolism. Future studies may help to better understand this possible role of H_2_S in DMD.

Until now, knowledge of the effects of H_2_S on skeletal muscles is limited even in animal models of DMD. Although various factors that liberate H_2_S after in vivo delivery have been described, their effectiveness in patients with DMD has not yet been evaluated. Unfortunately, systematic treatment with such compounds can be accompanied by the generation of reactive by-products, which can counteract the effect of H_2_S itself [163]. The cooperation of chemists and biologists is required to design and deeply characterize the new donors that will release this cytoprotective gas in a controlled way at a concentration comparable to endogenous H_2_S production.

## Data Availability

Not applicable.

## Abbreviations

AMPK: Adenosine 5′-monophosphate-activated protein kinase
BMD: Becker muscular dystrophy
CBS: Cystathionine β-synthase
CK: Creatine kinase
CSE: Cystathionine γ-lyase
DAPC: Dystrophin-associated protein complex
DGC: Dystrophin–glycoprotein complex
DMD: Duchenne muscular dystrophy
ECM: Extracellular matrix
HO-1: Heme oxygenase-1
LDH: Lactate dehydrogenase
3-MST: MPST: 3-mercaptopyruvate sulfurtransferase
NOS: Nitric oxide synthase
NRF2: Nuclear factor-erythroid 2-related factor 2
ROS: Reactive oxygen species
SCs: Satellite cells
